# CRA-1 Uncovers a Double-Strand Break-Dependent Pathway Promoting the Assembly of Central Region Proteins on Chromosome Axes During *C. elegans* Meiosis

**DOI:** 10.1371/journal.pgen.1000088

**Published:** 2008-06-06

**Authors:** Sarit Smolikov, Kristina Schild-Prüfert, Mónica P. Colaiácovo

**Affiliations:** Department of Genetics, Harvard Medical School, Boston, Massachusetts, United States of America; National Cancer Institute, United States of America

## Abstract

The synaptonemal complex (SC), a tripartite proteinaceous structure that forms between homologous chromosomes during meiosis, is crucial for faithful chromosome segregation. Here we identify CRA-1, a novel and conserved protein that is required for the assembly of the central region of the SC during *C. elegans* meiosis. In the absence of CRA-1, central region components fail to extensively localize onto chromosomes at early prophase and instead mostly surround the chromatin at this stage. Later in prophase, central region proteins polymerize along chromosome axes, but for the most part fail to connect the axes of paired homologous chromosomes. This defect results in an inability to stabilize homologous pairing interactions, altered double-strand break (DSB) repair progression, and a lack of chiasmata. Surprisingly, DSB formation and repair are required to promote the polymerization of the central region components along meiotic chromosome axes in *cra-1* mutants. In the absence of both CRA-1 and any one of the *C. elegans* homologs of SPO11, MRE11, RAD51, or MSH5, the polymerization observed along chromosome axes is perturbed, resulting in the formation of aggregates of the SC central region proteins. While radiation-induced DSBs rescue this polymerization in *cra-1; spo-11* mutants, they fail to do so in *cra-1; mre-11*, *cra-1; rad-51,* and *cra-1; msh-5* mutants. Taken together, our studies place CRA-1 as a key component in promoting the assembly of a tripartite SC structure. Moreover, they reveal a scenario in which DSB formation and repair can drive the polymerization of SC components along chromosome axes in *C. elegans*.

## Author Summary

Accurate meiotic chromosome segregation relies on homologous chromosome pairing, synapsis, and recombination. Although formation of the “zipper-like” structure known as the synaptonemal complex (SC) is critical for homologous chromosome synapsis in most sexually reproducing organisms, regulation of SC formation is still poorly understood. Previous studies revealed that whereas SC formation is dependent on the formation of double-strand breaks (DSBs) in yeast, plants, and mammals, it is DSB-independent in flies and nematodes. Here we introduce CRA-1, a novel and conserved protein required for the formation of the SC in the nematode *C. elegans*. In *cra-1* mutants, SC central region components for the most part fail to link homologous chromosome axes and stabilize homologous pairing interactions. As a result, crossover recombination is impaired and there is increased chromosome nondisjunction. Analysis of *cra-1* mutants also reveals that DSB formation and repair can promote the assembly of SC proteins along chromosome axes. Therefore, we propose that CRA-1 promotes a productive SC assembly, and demonstrate, in our analysis of *cra-1* mutants, an unanticipated interconnection between the recruitment of central region components onto chromosome axes and the recombination pathway in *C. elegans*.

## Introduction

Meiosis is a specialized cell division program required for the formation of haploid gametes, and is therefore essential for most sexually reproducing organisms. The reduction of the genome complement by half is achieved by following a single round of DNA replication with two consecutive rounds of chromosome segregation. A series of steps are undertaken to ensure the faithful segregation of chromosomes during meiosis. During the extended prophase of meiosis I, homologous chromosomes pair, and a proteinaceous structure known as the synaptonemal complex (SC) assembles and connects the axes of homologs throughout their full lengths. Chromosomes also undergo programmed meiotic DNA double-strand breaks (DSBs), a subset of which is repaired via reciprocal exchange between homologous nonsister chromatids. These crossovers, in collaboration with flanking cohesion between sister chromatids, result in physical attachments (chiasmata) between homologs that persist after SC disassembly. These chiasmata are critical for proper metaphase alignment and subsequent accurate segregation of homologs to opposite ends of the spindle upon onset of the first meiotic division. Therefore, DSB formation and productive SC polymerization, resulting in the stabilization of homologous interactions, are prerequisites for proper meiotic chromosome segregation. However, the coordination between these processes is not fully understood.

The SC is a tripartite structure comprised of a pair of lateral elements, consisting of proteins assembled along the axis of each homolog, and a central region, consisting of transverse filament proteins interconnecting homologous axes. The SC is highly conserved at the ultrastructural level and several of its structural components have been identified in yeast, flies, worms, plants, rats and mice [1],[2]. In *C. elegans*, the lateral element protein HIM-3 [3] loads onto chromosomes upon entry into meiosis at the transition zone (leptotene/zygotene), followed by the central region SYP (SYP-1, SYP-2, SYP-3) family of proteins which assemble to create the mature SC [4],[5],[6]. However, SC central region components have a proclivity for polymerization, as shown by their inappropriate assembly both along chromosome axes and between nonhomologous chromosomes in many organisms when meiosis is perturbed [6],[7],[8],[9],[10]. Therefore, SC polymerization has to be tightly regulated in order to result in homologous synapsis. This regulation is enforced throughout the establishment and the stabilization of pairing interactions between homologous chromosomes. Pairing interactions are initiated through the attachment of either telomeres or telomere proximal regions to the nuclear envelope and the tight clustering of chromosomes within a nuclear subdomain [9],[11]. In the case of *C. elegans*, this involves the homolog recognition regions or pairing center (PC) ends of chromosomes [12]. The stabilization of these pairing interactions requires the formation of a functional SC structure [4],[5],[6]. These processes are further regulated, as is evident from recent studies in *C. elegans* of proteins such as HTP-1 and SYP-3. HTP-1 is a HORMA domain protein essential for coordinating the pairing and synapsis necessary for homologous synapsis [7],[8]. SYP-3 restricts central region formation to coupled homologous axes [6].

Studies of SC function have revealed that SC formation between homologous chromosomes plays a key role in the normal progression of meiotic recombination. Mutants that fail to form the central region of the SC in yeast, plants and mice have reduced crossover levels [13],[14],[15]. Furthermore, in *C. elegans* and *D. melanogaster*, lack of SC assembly results in the absence of crossovers [4],[5],[6],[16].

However, while either homologous chromosome synapsis or the localized assembly of synapsis-related proteins at crossover sites is a prerequisite for the maturation of crossover events in many species, the requirement of DSB formation to achieve synapsis has not seemed so widely conserved. In *S. cerevisiae*, *A. thaliana* and mouse mutants that lack Spo11, a conserved topoisomerase-like protein required for the formation of meiotic DSBs [17],[18], levels of SC formation are either dramatically reduced [15],[19] or the SC is frequently assembled between nonhomologous chromosomes [20],[21]. In contrast, *spo11* mutants in both *C. elegans* and *D. melanogaster* do not affect SC formation, although they do lack chiasmata [22],[23]. Therefore, it has been proposed that while SC formation is DSB-dependent in yeast, plants and mammals, it is DSB-independent in *C. elegans* and *Drosophila*.

In the current study, we identify and analyze CRA-1, a tetratricopeptide repeat (TPR) domain-containing protein conserved throughout multicellular organisms. Our analysis of CRA-1 function has revealed an unanticipated requirement for both meiotic DSB formation and repair in driving the polymerization of central region components of the SC along chromosome axes in *C. elegans.* We show that in *cra-1* mutants, extensive localization of SC central region components along chromosome axes is delayed and fails to efficiently connect homologous axes. This results in defects in the stabilization of pairing interactions, progression of meiotic recombination and chiasma formation. Moreover, CRA-1 acts downstream from both axis-associated and central region components of the SC, therefore identifying a new class of proteins required for proper SC assembly in *C. elegans*. Surprisingly, the lack of DSB formation in *cra-1; spo-11* mutants impairs the polymerization of central region components of the SC along chromosome axes and alters chromosome organization. However, both this polymerization and chromosome redispersal can be rescued by the induction of exogenous DSBs. A similar block to the polymerization of central region components along chromosome axes is observed in *cra-1* mutants combined with *mre-11*, *rad-51* or *msh-5* mutations, but this cannot be rescued by ionizing radiation-induced DSBs, suggesting that progression of DSB repair is required to promote this polymerization. Altogether, our analysis identifies CRA-1 as a new component involved in promoting functional chromosome synapsis and reveals a novel context in which the recruitment of central region components onto chromosome axes and the recombination pathway are interconnected in *C. elegans*.

## Results

### CRA-1 Is a Conserved TPR Containing Protein

**Figure 1:**
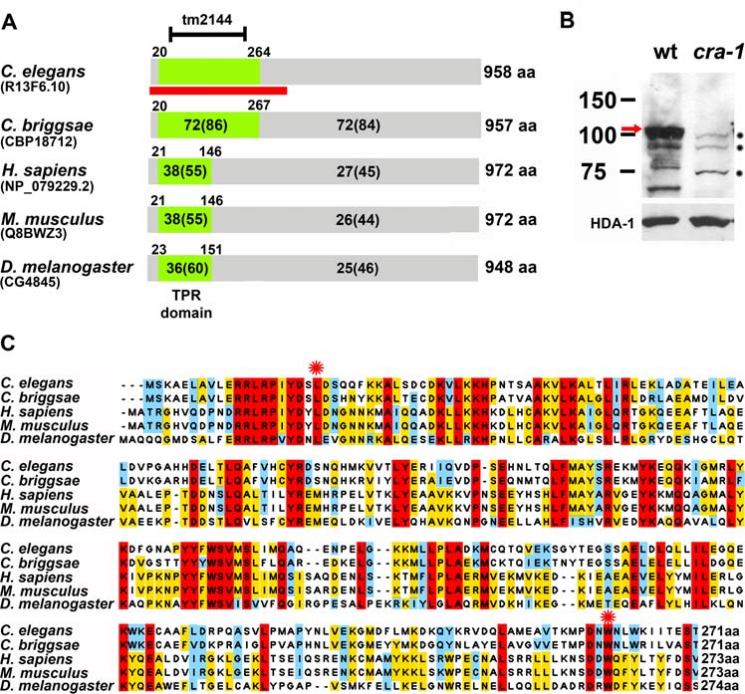
CRA-1 Protein Structure, Conservation and Expression in Wild Type and *cra-1(tm2144)* Mutants. (A) Schematic representation of the predicted CRA-1 protein in *C. elegans*, and its presumed orthologs in *C. briggsae*, *H. sapiens*, *M. musculus* and *D. melanogaster*. The region deleted in the *tm2144* mutant allele is indicated (codons 49 through 234). TPR domains, with start and end points identified through InterProScan [69], are depicted in green. Percent identity and similarity (in parentheses) are indicated representing both the degree of conservation exclusively in the TPR domain as well as throughout the entire protein. The red bar indicates the region used for generating the anti-CRA-1 antibody. (B) Western blot analysis comparing wild type and *cra-1(tm2144)* mutant lysates probed with anti-CRA-1 and anti-histone deacetylase 1 (HDA-1; loading control) antibodies. A wild type specific band corresponding to the expected 109 kDa CRA-1 protein is observed (red arrow). Asterisks indicate cross-reacting bands. (C) Sequence alignment of the first 271–274 amino acids of CRA-1 and indicated orthologs. Red asterisks delimit the region defined as a TPR domain in *C. elegans*. Amino acids conserved in all proteins are highlighted in red. Amino acids conserved in at least three proteins are highlighted in yellow. Highly related amino acids are shaded in blue. Image was generated using ClustalW and Jalview programs [70].

*cra-1* was identified in an RNA interference (RNAi)-mediated functional genomics screen for meiotic genes (see Materials and Methods). The *cra-1(tm2144)* mutant carries an out-of-frame 753 base pair deletion encompassing most of its predicted TPR domain (Figure 1A). Genetic analysis of *tm2144* revealed that it is a null allele of *cra-1* (see Materials and Methods). Furthermore, the 109 kDa band corresponding to CRA-1 observed in lysates prepared from wild type worms is absent in lysates from equal numbers of *cra-1(tm2144)* worms, reflecting a lack of CRA-1 protein in these mutants (Figure 1B).

BLAST database searches indicated that CRA-1 is conserved across multicellular organisms (Figure 1A, C). CRA-1 has clear orthologs in both *C. briggsae* and *C. remanei* and shares a high percentage of similarity throughout its full length with proteins of unknown function in *H. sapiens*, *M. musculus* and *D. melanogaster*. Interestingly, expression data indicate that the human and mouse homologs are expressed in testis and that the fly homolog is specifically enriched in both ovary and testis 24,25,26. Slightly higher identity and similarity levels are shared throughout the TPR domain, suggesting functional specialization of the TPR motif. This TPR domain is a protein-protein interaction domain found in various proteins serving as scaffolds for the assembly of multiprotein complexes and participating in various biological processes including cell cycle regulation and transcriptional control ([27],[28]; for review [29]). Strikingly, both the length of CRA-1 and the N-terminal positioning of the TPR domain are conserved. No additional domains are observed in CRA-1.

### Chromosome Synapsis and Chiasma Formation Are Defective in *cra-1* Mutants

Analysis of *cra-1(tm2144)* mutants revealed severe defects in meiotic chromosome segregation. In *C. elegans*, meiotic chromosome nondisjunction results in an increase in inviable aneuploid embryos accompanied by a high incidence of males (Him) phenotype among the surviving progeny produced by hermaphrodite worms. *cra-1(tm2144)* mutants exhibited a very high level of embryonic lethality (99.74%, n = 7018) accompanied by larval lethality (61%). In contrast to wild type, where hermaphrodites (XX) lay male (XO) progeny at a very low frequency (0.2%; [30]), a likely Him phenotype was observed among viable *cra-1(tm2144)* progeny, although an exact assessment of the severity of the Him phenotype was made difficult by the very low levels of progeny reaching adulthood (7/7018 viable adults of which 1/7 were males). Therefore, altogether our data are consistent with an increase in meiotic chromosome nondisjunction in *cra-1* mutants.

**Figure 2:**
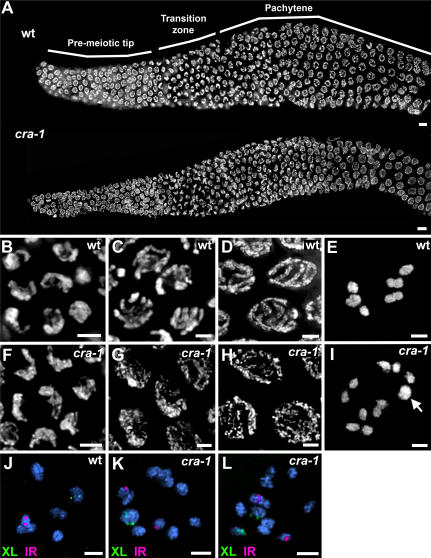
Chromosome Morphogenesis and Nuclear Organization During Meiotic Prophase Are Altered in *cra-1* Mutants. (A) Low magnification images of DAPI-stained nuclei of whole-mount gonads from age-matched wild type and *cra-1(tm2144)* adult hermaphrodites. Progression throughout early- and mid-prophase is observed from left to right. (B–I) High magnification images of DAPI-stained nuclei taken from the transition zone (B, F), early pachytene (C, G), late pachytene (D, H) and diakinesis (E, I) of wild type (B–E) and *cra-1(tm2144)* (F–I) depicting the defects observed in chromosome morphogenesis and nuclear organization. Arrow indicates a bivalent in the *cra-1(tm2144)* diakinesis oocyte in (I). (J–L) High magnification images of diakinesis oocytes stained with DAPI (blue) and hybridized with FISH probes targeting the left end of the X chromosome (green) and the right end of chromosome I (red) in wild type (J) and *cra-1(tm2144)* mutants (K–L). Bars, 5 µm (A) and 2 µm (B–L).

Cytological analysis revealed that *cra-1(tm2144)* mutants exhibit defects in chromosome synapsis. In *cra-1(tm2144)* mutants, chromosomes entered meiosis in the transition zone (leptotene/zygotene), acquiring a polarized spatial organization characteristic of this stage (Figure 2A–B, F). However, while in wild type, chromosomes then redispersed throughout the nuclear periphery upon entrance into pachytene (Figure 2A, C), in *cra-1(tm2144)* mutants, chromosomes failed to redisperse and remained in a clustered configuration until what corresponds to mid-pachytene in wild type (Figure 2A, G). Furthermore, once chromosomes redispersed, instead of the thick and parallel DAPI-stained tracks characteristic of synapsed chromosomes at this stage in wild type (Figure 2A, D), mostly thin and unaligned tracks were apparent (Figure 2A, H). Interestingly, the defect in the timing of chromosome redispersal observed in *cra-1* mutants is different from that observed in mutants lacking either axis or central region components of the SC. Specifically, *him-3* and *htp-1* null mutants result in either a lack or a reduced number of nuclei in a polarized spatial organization [7],[8],[31], while in *syp-1*, *syp-2* and *syp-3* null mutants, chromosomes acquire a clustered configuration, but redisperse much later in pachytene [4],[5],[6].

Further cytological analysis revealed that autosomes lack chiasma formation more frequently than the X chromosome in *cra-1(tm2144)* mutants. In wild type diakinesis oocytes, chromosomes condense and six DAPI staining bodies are observed, corresponding to six pairs of attached homologous chromosomes (bivalents; Figure 2E). However, in *cra-1(tm2144)* mutants, oocytes with either 11 or 12 DAPI staining bodies were observed (39% and 54 %, respectively, n = 119; Figure 2I). This indicates that, in more than half of the *cra-1(tm2144)* oocytes, all six homologous chromosomes fail to form chiasmata, while in the remaining oocytes one of the six pairs of homologous chromosomes forms a chiasma. To determine whether the single successful chiasma involves a specific chromosome or occurs at random, diakinesis oocytes were examined utilizing fluorescence in situ hybridization (FISH) probes for chromosomes I and X. In wild type, each one of these probes recognizes unique individual bivalents (Figure 2J). In *cra-1(tm2144)* mutants, while the bivalent rarely corresponded to chromosome I (1/29), it frequently corresponded to the X chromosome (16/29; Figure 2K–L), demonstrating that successful chiasma formation frequently involves the X. This is further supported by the reduction in crossover frequencies observed in *cra-1(tm2144)* mutants. Although crossover recombination was reduced along both chromosomes V and X (8.5% and 67% of wild type levels, respectively), this reduction was much stronger on the autosomes than on the X chromosome (p<0.0001 and p = 0.1166, respectively, by the two-tailed Fisher's Exact Test, 95% C.I.; Table 1). Taken together, these results suggest that the autosomes are more stringent in their requirement of CRA-1 function for chiasma formation.

**Table 1:** Crossover Rcombination Is Reduced in *cra-1* Mutants.

Genotype	Chromosome	No. of recombinants	Total No. of embryos examined	Map distance (cM)
*cra-1/+*	V^a^	36	88	40.9
*cra-1/cra-1*	V^a^	2	57	3.5
*cra-1/+*	X^b^	27	72	37.5
*cra-1/cra-1*	X^b^	20	79	25.3

a SNPs used for chromosome V: pkP5076 and snp_Y17D7B.

b SNPs used for the X chromosome: pkP6143 and uCE6-1554.

### CRA-1 Is Required for the Stabilization of Homologous Chromosome Pairing Interactions

**Figure 3:**
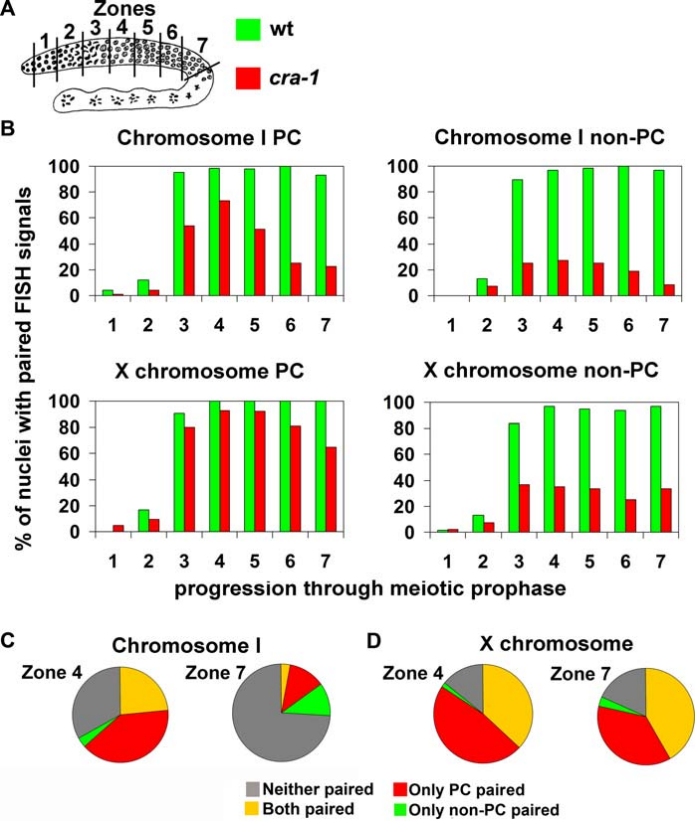
CRA-1 Acts Downstream of Homology Recognition in the Stabilization of Homologous Pairing Interactions. (A) Diagram of a *C. elegans* germline indicating the position of the zones scored for a time-course analysis of homologous pairing. The color codes represent the genotypes examined in (B). (B) Graphs depict the percentage of nuclei carrying paired homologous chromosomes (y-axis) within each zone along the germline (x-axis). The regions being recognized by the FISH probes are indicated above the graphs. Each graph depicts data collected from whole mount germlines examined with a single FISH probe. (C–D) Analysis of pairing simultaneously at both ends of either chromosome I (C) or the X chromosome (D) in *cra-1* mutants. Pie charts depict the percentage of nuclei in zones 4 and 7 where chromosomes are paired at both the pairing center (PC) and nonpairing center (NPC) ends or just at either single end. Data was collected from FISH performed on whole mounts.

Quantitative analysis of chromosome pairing levels through FISH suggests that defects in chiasma formation stem from the impaired stabilization of homologous pairing interactions in *cra-1(tm2144)* mutants (Figure 3). Furthermore, while homologous pairing is decreased in *cra-1(tm2144)* mutants compared to wild type for all loci tested, the levels of change differ considerably between loci on different chromosomes (Figure 3A–B; Table S1). When assaying for pairing at the PC ends of chromosomes, the maximum level of pairing achieved in early pachytene (zone 4) decreased from 98% to 73% for chromosome I and from 100% to 92% for the X chromosome. At the end of pachytene (zone 7), pairing levels in *cra-1(tm2144)* dropped from 92% to 23% and 100% to 65% for chromosomes I and X, respectively (Figure 3B). Furthermore, when pairing interactions were examined for both ends of a single chromosome simultaneously, an additional observation was made. While both chromosomes I and the X achieved high levels of pairing at both PC and non-PC ends initially (Figure 3C–D, zone 4, yellow), this pairing interaction was stabilized mostly for the X chromosome (Figure 3C–D, zone 7, yellow). These results indicate that the stabilization of chromosome pairing interactions depends on CRA-1, and further suggest that the X chromosome is not as stringent in its requirement for CRA-1 as are the autosomes. Therefore, the higher frequency of chiasma formation observed for the X chromosome is strongly correlated to the higher percentage of stable pairing achieved by this chromosome as compared to the autosomes. Interestingly, differences in chromosomal pairing between autosomes and the X chromosomes are not unique to *cra-1* mutants. Both *him-3* hypomorphs [31],[32], which can partly assemble the SC central region, and *htp-1* mutants [7],[8] show severely reduced chromosome pairing on autosomes accompanied by wild type levels of pairing for the X chromosome. This may result from the X chromosome being more efficient in SC assembly in a situation in which SC assembly is perturbed, but not eliminated.

When comparing pairing levels between the PC and non-PC ends of chromosomes, we also determined that in *cra-1(tm2144)* mutants, pairing at the PC end is significantly higher than at the non-PC end. In *cra-1(tm2144)* mutants, the maximum levels of pairing achieved at the non-PC ends (zone 4) were 27% and 35%, respectively, for chromosomes I and X, which are 2.7- and 2.6-fold lower, respectively, than the levels of pairing at the PC ends (Figure 3B). This further indicates that synapsis-independent pairing, which is PC end-mediated in *C. elegans* [4],[12], can occur in *cra-1(tm2144)*, but that these pairing interactions cannot be stabilized. In this respect, the pairing defects observed in *cra-1(tm2144)* mutants resemble those seen in the *syp-1, syp-2* and *syp-3* null mutants, which lack assembly of the central region of the SC, and differ significantly from mutants in genes essential for lateral element formation, which are impaired for the initial establishment of pairing [4],[5],[6],[8],[31],[33].

### Progression of Meiotic Recombination Is Impaired in *cra-1* Mutants

**Figure 4:**
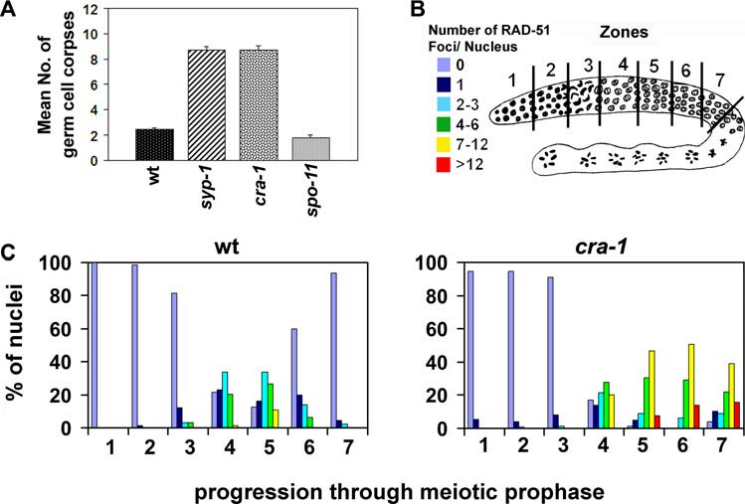
Progression of Meiotic Recombination is Impaired in *cra-1* Mutants. (A) Graphic representation of the mean number of germ cell corpses with standard error bars, indicating the elevated germ cell apoptosis observed in *cra-1* mutants compared to either wild type or *spo-11* mutants. (B) Diagram of a *C. elegans* germline depicting the seven zones throughout which RAD-51 foci were quantitated. Levels of RAD-51 foci are indicated by the color code. (C) Histograms depict the quantitation of RAD-51 foci in germlines of the indicated genotypes. The percent of nuclei observed for each category indicated by the color code (y-axis) are depicted for each zone along the germline (x-axis).

**Figure 5:**
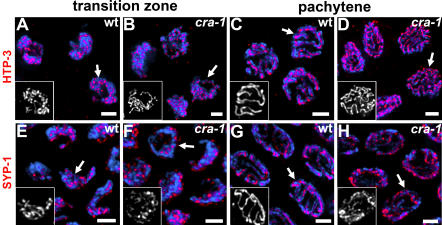
CRA-1 is Required for SC Central Region Formation. (A–H) High magnification images of nuclei from the transition zone and late pachytene in wild type and *cra-1* mutants. DAPI-stained chromosomes are immunostained with anti-HTP-3 to observe the lateral element (A–D) and anti-SYP-1 to visualize central region formation of the SC (E–H). Insets represent antibody only images of nuclei indicated by the arrows to facilitate the observation of both the continuity and distribution of the observed signals. In *cra-1* mutants, HTP-3 is observed localizing continuously along chromosomes at the transition zone and pachytene, indicating normal lateral element formation. In contrast, the SYP-1 signal is observed mostly surrounding chromosomes instead of being directly localized onto chromosomes at the transition zone and is observed in variable levels along unsynapsed chromosomes at pachytene. Bars, 2 µm.

**Figure 6:**
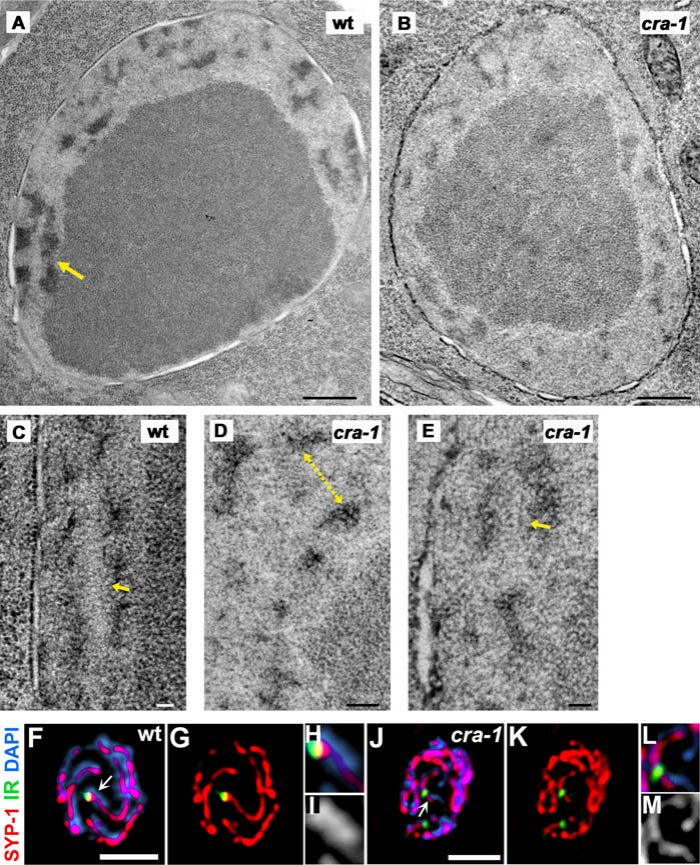
A Low Frequency of Synapsis in *cra-1* Mutants. (A–E) TEM images of late pachytene nuclei in wild type and *cra-1* germlines. (A) Equatorial section of a wild type late pachytene nucleus. The large dark body slightly off-center is the nucleolus surrounded by SC stretches. The SC stretches are visible as faint zipper-like tracks flanked by electron-dense patches of chromatin (indicated by a yellow arrow). (B) Equatorial section of a *cra-1* late pachytene nucleus, in which chromatin patches are observed surrounding the nucleolus and SC stretches are absent. (C) High magnification of a stretch of SC from a different wild type late pachytene nucleus. The distance between electron-dense chromatin patches arranged in parallel was approximately 100nm; the yellow arrow indicates a region where transverse filaments are more apparent. (D) High magnification of chromatin patches observed in a different *cra-1* late pachytene nucleus. Distances between chromatin patches occasionally observed in a parallel arrangement in *cra-1* mutants were measured as indicated by the yellow dotted arrow. (E) High magnification of an occasional SC stretch observed in a different *cra-1* late pachytene nucleus. The yellow arrow indicates a region where transverse filaments are more apparent and measurements confirmed the approximately 100nm distance in these SC stretches. Bars, 500 nm (A–B), 100 nm (C and E), and 200 nm (D). (F–M) High magnification of a single pachytene nucleus simultaneously assessing pairing at the PC end of chromosome I (IR; green) by FISH and observing the chromosomal association of SYP-1 (red) by immunostaining. Panels H–I and L–M are higher magnification images of regions indicated by arrows respectively in F and J. In wild type (F–I), thick DAPI signals flank the SYP-1 signal at the paired loci, indicating homologous synapsis. In contrast, in *cra-1* mutants (J–M), SYP-1 is mainly observed localizing to very thin DAPI-stained chromatin tracks at unpaired loci, suggesting that SYP-1 is associated along unsynapsed chromosomes and that synapsis is infrequent. Bars, 2 µm.

Unrepaired meiotic DSBs have been shown to result in an increase in germ cell corpses due to activation of a late pachytene DNA damage checkpoint [34]. Therefore, we examined the levels of germ cell death in *cra-1(tm2144)* mutants to determine whether the lack of stabilization of homologous pairing interactions would correlate with unrepaired meiotic DSBs. In *cra-1(tm2144)* mutants, similar to *syp* mutants, a 3.6-fold and 4.8-fold increase in apoptosis levels was observed compared to wild type and to DSB-defective *spo-11* mutants, respectively (Figure 4A; [4],[5],[6]). Therefore, elevated germ cell apoptosis in *cra-1(tm2144)* mutants suggests an altered progression of meiotic recombination, resulting in the presence of unrepaired recombination intermediates in late pachytene.

To directly examine the progression of meiotic recombination, a quantitative analysis of nuclei stained with α-RAD-51 was performed (Figure 4B–C). This antibody marks sites undergoing DSB repair, as RAD-51 is needed for the strand exchange step of recombination [5],[35],[36]. In wild type, levels of RAD-51 foci increased upon entrance to the transition zone (zone 3), peaked at early to mid-pachytene (zone 5) and disappeared by the end of pachytene (zone 7). In *cra-1(tm2144)* mutants, levels of RAD-51 foci increased with wild type kinetics upon entrance into meiosis, suggesting that the initiation of meiotic recombination is not altered in this mutant. However, levels of RAD-51 foci were higher than wild type upon entrance into pachytene and remained elevated throughout late pachytene (zones 4–7), similar to *syp-1*, *syp-2* and *syp-3* null mutants [4],[5],[6]. These results suggest that progression of meiotic recombination is impaired in *cra-1(tm2144)* mutants. Altogether, our results point to the essential role of CRA-1 in DSB repair, likely executed through its function in ensuring the stabilization of homologous pairing, which in turn is critical for the progression of meiotic recombination.

### CRA-1 Is Required for the Assembly of the Central Region of the SC

To address whether the defective stabilization of homologous pairing observed in *cra-1(tm2144)* mutants results from defects in proper SC assembly, we examined the localization of SC-associated components throughout meiotic prophase in *cra-1(tm2144)* mutants. We found no difference compared to wild type in the timing, pattern or levels of localization of axis-associated proteins, such as the axial element components HIM-3 and HTP-3 and the meiosis-specific cohesin REC-8 (Figure 5A–D and data not shown). As exemplified by α-HTP-3 staining, continuous stretches of HTP-3 formed along chromosomes upon entrance into meiosis (Figure 5A–B), and were observed along synapsed chromosome axes in wild type and along mostly unsynapsed chromosome axes in *cra-1(tm2144)* mutants throughout late pachytene (Figure 5C–D). Therefore, axis morphogenesis is not detectably altered in *cra-1* mutants.

In contrast, chromosomal localization of all three central region components of the SC (SYP-1, SYP-2 and SYP-3) is impaired in *cra-1(tm2144)* mutants (Figure 5E–H and data not shown). In the transition zone nuclei, SYP-1 failed to form the continuous linear tracks observed along chromosomes in wild type. Instead, SYP-1 mostly surrounded chromosomes and occasionally formed foci or short stretches throughout chromatin tracks (Figure 5E–F). Co-immunostaining of *cra-1(tm2144)* gonads with α-HTP-3 and α-SYP-1 further demonstrated that axis morphogenesis occurs normally, whereas SYP-1 fails to associate with chromatin properly, acquiring, instead, mostly a peripheral localization, which persists until chromosomes redisperse (Figure S1). In pachytene nuclei, instead of the continuous localization of SYP-1 observed between paired and aligned homologs in wild type (Figure 5G), SYP-1 associated with both the thinner and occasional thick DAPI-stained tracks apparent in *cra-1(tm2144)* mutants (Figure 5H). Furthermore, not all tracks showed a similar intensity of SYP-1 signal, suggesting an uneven distribution of SYP-1 throughout chromatin tracks in *cra-1(tm2144)* mutants. Finally, disassembly of chromosome axes-associated central region components in *cra-1(tm2144)* mutants occurred with kinetics similar to that of SC disassembly in wild type during late pachytene and diplotene. However, during diakinesis in *cra-1(tm2144)* mutants, instead of SYP-1 being restricted to the short arms of all six bivalents, as is observed in wild type, or being localized extensively throughout univalents, as is observed in recombination mutants, only a single focus or stretch of SYP-1 was apparent, frequently at the ends of univalents (Figure S2A–C and [37]).

Transmission electron microscopy (TEM) analysis of late pachytene nuclei in both wild type and *cra-1* mutant germlines also supports a role for CRA-1 in SC formation. SC stretches were observed in 64% of the wild type nuclei in late pachytene (an average of 1.2 SC stretches per nucleus; n = 55), whereas dispersed patches of electron dense chromatin, not associated with any detectable SC, were observed for 86% of the *cra-1* nuclei at this stage (n = 29; Figure 6A–E). However, while SC stretches, where an ordered array of transverse filaments is located between parallel masses of electron-dense chromatin (Figure 6A, C) were more frequent in wild type, 14% of the *cra-1* nuclei had 1 SC stretch (Figure 6B, D–E). Therefore, this analysis, coupled to the observation of occasional thick parallel DAPI-stained tracks in *cra-1(tm2144)* late pachytene nuclei (Figure 2H) suggest that some synapsis occurs in the *cra-1(tm2144)* mutant.

To examine whether the synapsis observed in *cra-1* mutants involves homologous or nonhomologous chromosomes, we monitored pairing on chromosome I by FISH in combination with immunostaining for a marker of the central region of the SC (SYP-1) in late pachytene nuclei. In wild type nuclei, paired FISH signals coincide with sites bearing SYP-1 flanked by thick DAPI signals, corresponding to a pair of synapsed homologous chromosomes (Figure 6F–I). In contrast, in *cra-1(tm2144)* late pachytene nuclei, FISH signals were frequently unpaired and localized to thin unsynapsed DAPI-stained tracks (85.5% and 88%, respectively, for the PC and non-PC ends of chromosome I; n = 65/76 and n = 64/73; Figure 6J–M). However, occasionally in *cra-1* pachytene nuclei, paired FISH signals for either the PC or non-PC ends of chromosome I (10.5% and 8%, respectively), and unpaired FISH signals (4% at either PC or non-PC ends) coincided with sites where thick DAPI signals flanked SYP-1. Taken together, these results suggest that both homologous and nonhomologous synapsis occur, albeit infrequently, in *cra-1* mutants.

### CRA-1 Acts Downstream of SC-Associated Proteins

In order to establish the epistatic relationship between CRA-1 and components acting at various steps of the SC assembly pathway, we created double mutants containing *cra-1(tm2144)* in combination with *him-3* (acting during axis morphogenesis; [3]), *htp-1* (acting in the coordination of pairing and synapsis;[7],[8]) or *syp-3* (acting in central region formation; [6]).

In *him-3* null mutants, chromosomes fail to acquire the polarized spatial organization characteristic of the transition zone and instead remain dispersed and unsynapsed throughout prophase [3],[31]. The chromosome morphology of *cra-1(tm2144); him-3* double mutants was indistinguishable from that of *him-3* single mutants (Figure S3A–B and data not shown). This suggests that CRA-1 acts downstream of HIM-3, further supporting the observation that CRA-1 is not required for axis morphogenesis.

*cra-1(tm2144); htp-1* double mutants exhibited a combination of phenotypes observed in each of the corresponding single mutants (Figure S3C–D). Specifically, in *cra-1(tm2144); htp-1* mutants, nuclei with chromosomes in a clustered organization were infrequent and chromosome redispersal occurred prematurely, similar to what is observed for *htp-1* mutants. However, instead of the extensive nonhomologous synapsis observed in *htp-1* mutants (Figure S3C and [7],[8]), chromosomes were unsynapsed as in *cra-1(tm2144)* mutants. Interestingly, SYP-1 chromosomal association was reduced more severely than in *cra-1* mutants (Figure S3D). These results suggest that CRA-1 and HTP-1 may regulate different steps required for SC assembly, and that these steps occur at different times during meiotic prophase.

In *syp-3* null mutants, chromosomes persist in a polarized nuclear organization until late pachytene, therefore exiting this configuration later than what is observed in *cra-1(tm2144)* mutants [6]. *cra-1(tm2144); syp-3* double mutants were indistinguishable from *syp-3* mutants, both in the extent of nuclei with chromosomes in a transition zone-like morphology and in the lack of SYP-1 chromosomal localization (Figure S3E–F). To ensure that this outcome could be extrapolated to other components of the central region of the SC, we examined a *cra-1(tm2144)*; *syp-2* double mutant. As expected, given that all three SYP proteins are interdependent for their chromosomal localization [5],[6], and all three *syp* null mutants show similar chromosome organization defects, the same result was obtained in a *cra-1(tm2144)*; *syp-2* double mutant (data not shown).

Altogether, these results imply that CRA-1 acts downstream of the SYP proteins, where CRA-1 executes its function in a SYP-dependent manner. Moreover, these results further suggest that CRA-1 is not required for synapsis-independent pairing, homology sorting, or the initiation of loading of SC central region components. Instead, it is essential for coordinating the accurate assembly of central region proteins into a functional structure.

### Polymerization of Central Region Components of the SC Along Chromosome Axes Is Dependent on DSB Formation and Repair in *cra-1* Mutants

**Figure 7:**
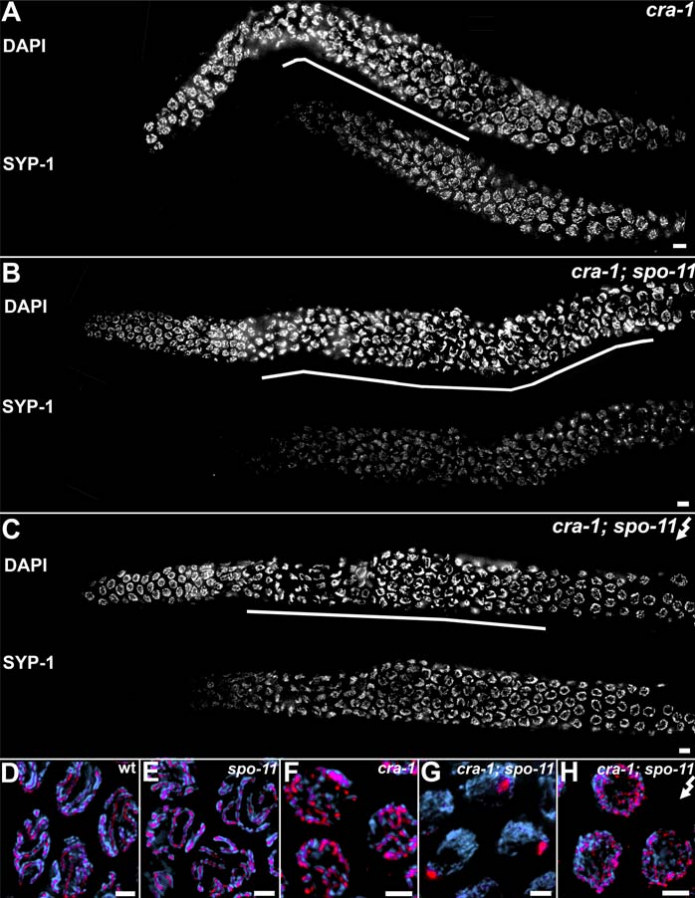
DSB Formation Promotes Polymerization of Central Region Components of the SC Along Chromosome Axes in *cra-1* Mutants. (A–C) Low magnification images of DAPI-stained nuclei of whole-mount gonads from age-matched *cra-1* (A), *cra-1; spo-11* (B), and 8 hours post-irradiation *cra-1; spo-11* (C) worms, immunostained with anti-SYP-1. Lines below DAPI-stained gonads indicate length throughout which nuclei persist with chromosomes in a polarized organization. (D–H) High magnification images taken from late pachytene nuclei of wild type (D), *spo-11* (E), *cra-1*(F), *cra-1; spo-11* (G), and 8 hours post-irradiation *cra-1; spo-11* (H) worms. Deletion of *spo-11* in the background of *cra-1* results in the formation of SYP-1 aggregates (B, G). This can be alleviated by introducing exogenous DSBs (C, H). Nuclei are co-stained with DAPI (blue) and anti-SYP-1 (red) (D–H). Bars, 5 µm (A–C) and 2 µm (D–H).

Given that the kinetics of chromosome redispersal observed in *cra-1* mutants are distinct from those observed in mutants for other components implicated in chromosome synapsis, we examined whether the formation of DSBs could play a role in driving these changes in chromosome configuration. In *spo-11* mutants, SC formation and chromosome morphogenesis throughout both the transition zone and pachytene are indistinguishable from wild type (Figure 7D–E and [23]). However, *spo-11* mutants lack chiasmata, since chromosomes fail to undergo crossover recombination. As a result, 12 univalents are observed in diakinesis oocytes (Figure S2B and [23]). Surprisingly, in *cra-1(tm2144);spo-11* double mutants, chromosomes no longer exited from a clustered organization at what corresponds to mid-pachytene in wild type. Instead, they persisted in a clustered configuration through late pachytene, which is similar to what is observed in *syp* null mutants (Figure 7A–B). Even more significantly, the association of central region components with chromosome axes was severely perturbed. Specifically, very low levels of SYP-1 localized to chromosomes in transition zone nuclei (Figure 7B). As nuclei entered the equivalent to mid-pachytene in wild type, most of the SYP-1 antibody signal was observed as a single large aggregate in every nucleus (Figure 7B, G). These large aggregates persisted throughout diakinesis, where they were associated with chromosomes in 83% (n = 23) of the oocytes examined (Figure S2D). Similar results were observed in *cra-1(RNAi); spo-11* mutants, suggesting that these observations were not specific to the *cra-1* allele being utilized in these studies (Figure S2G). Moreover, analysis of late pachytene nuclei in *cra-1(tm2144);spo-11* mutants (Figure S4) co-immunostained with α-HTP-3 and α-SYP-1, revealed that HTP-3 localized continuously throughout chromosome axes while SYP-1 was mostly restricted to aggregates. This suggested that the SYP-1 aggregates are not a result of impaired axis formation. Finally, the localization of SYP-1 along unsynapsed chromosomes in *cra-1(tm2144)* mutants is also observed in *syp-3(me42)* mutants [6]. However, in contrast to that observed in *cra-1(tm2144);spo-11* mutants, this localization pattern was not altered in *syp-3(me42); spo-11* double mutants (Figure S5). Taken together, these results suggest that the extensive localization of central region components observed throughout chromosome axes in *cra-1* mutants is DSB-dependent and specific to the lack of CRA-1 function.

We next examined whether the impaired localization of SYP-1 observed in *cra-1(tm2144); spo-11* mutants is due to the lack of DSB formation, as opposed to the loss of other potential functions provided by SPO-11 in meiosis. To this end, we examined SYP-1 localization upon induction of DSBs by γ-irradiation. Strikingly, at one hour post-irradiation most SYP-1 aggregates were no longer apparent. Instead, elongated tracks of SYP-1 staining were observed along unsynapsed chromosomes, resembling the pattern observed in *cra-1(tm2144)* single mutants (data not shown). These SYP-1 tracks were still present 8 hours post-irradiation, at a time when RAD-51 foci are no longer apparent, suggesting that repair of induced DSBs is complete (Figure 7C, H and data not shown). These results imply that while spreading of SYP-1 throughout chromosome axes is DSB-dependent, once this spreading occurs, it is maintained in a SPO-11-independent fashion. Moreover, rescue of extensive loading of SYP-1 throughout chromosome axes by γ-irradiating *cra-1; spo-11* double mutants correlated with a shortening of the extended transition zone. Specifically, chromosomes persisted in a polarized configuration until zone 7 (late pachytene) in *cra-1; spo-11* germlines (n = 3 germlines). In contrast, in γ-irradiated *cra-1; spo-11* germlines (n = 5), chromosomes persisted in this configuration only until zone 5 (early-mid pachytene), similar to the pattern observed in *cra-1* germlines (n = 5). This further supports a model in which the association of SYP proteins, even if not resulting in the coupling of homologous axes, may trigger chromosome redispersal [8],[38].

Further analysis also revealed that while elongated tracks of SYP-1 staining were observed shortly after DSB formation in nuclei in early and mid-prophase, in contrast, the appearance of SYP-1 stained chromatin tracks is delayed in diakinesis oocytes. SYP-1 aggregates started to disappear 8 hours post-irradiation, but were only completely absent 16 hours post-irradiation in diakinesis oocytes (Figure S2E–F). Given that nuclei move along the germline as they progress through prophase, this data suggests that DSB-dependent SYP-1 localization throughout chromosome axes may only occur in meiocytes present earlier in prophase which will be progressively displaced and only reach diakinesis several hours later. Therefore the capacity to drive the extensive polymerization of central region components of the SC via DSB formation may be confined to earlier stages in prophase.

**Figure 8:**
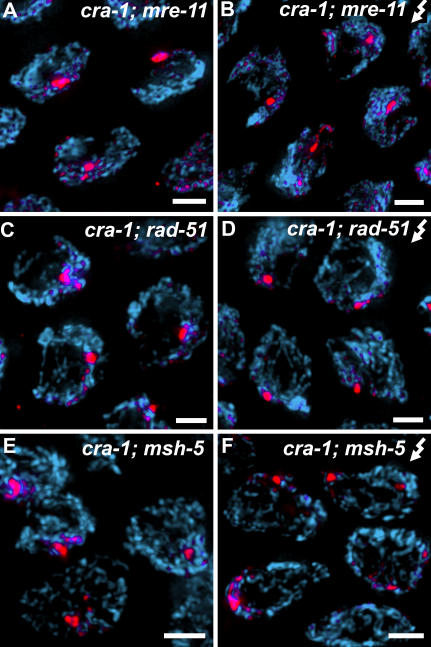
DSB Repair Proteins are Required for the Polymerization of Central Region Components of the SC Along Chromosome Axes in *cra-1* Mutants. (A–F) High magnification images of late pachytene nuclei from age-matched *cra-1; mre-11*, *cra-1; rad-51* and *cra-1; msh-5* mutant hermaphrodites which have not been irradiated (A, C, E), and 8 hours post-irradiation (B, D, F). SYP-1 aggregates are observed in *cra-1; mre-11, cra-1; rad-51* and *cra-1; msh-5* double mutants (A, C, E), suggesting that DSB repair proteins are required to prevent SYP-1 aggregation. Introducing exogenous DSBs does not alleviate this phenotype (B, D, F). Nuclei are co-stained with DAPI (blue) and anti-SYP-1 (red). Bars, 2 µm.

Finally, we examined if DSB formation is the sole requirement for SYP-1 assembly along chromosome axes in *cra-1* mutants or if proceeding through DSB repair is also required for this assembly. In order to test this possibility we created double mutants containing *cra-1(tm2144)* and *mre-11* (acting early in recombination, mainly in DSB processing; [39]), *rad-51* (acting in DNA strand exchange during DSB repair; [36]) or *msh-5* (acting later in recombination to promote crossovers; [40]). Like *spo-11* mutants, *mre-11*, *rad-51* and *msh-5* single mutants show normal SC assembly, but lack chiasmata [5],[35],[39],[40]. In contrast, in *cra-1; mre-11*, *cra-1; rad-51* and *cra-1; msh-5* double mutants, chromosomes persisted in a clustered configuration through late pachytene accompanied by the formation of SYP-1 aggregates, as is seen in *cra-1; spo-11* double mutants (Figure 8A, C, E). Interestingly, SYP-1 aggregates appeared later in *cra-1; msh-5* double mutants, than they do in *cra-1; spo-11, cra-1; rad-51* or *cra-1; mre-11* double mutants, correlating with a role downstream of DSB formation or processing. Therefore, abrogating DNA repair once DSBs are formed results in phenotypes similar to those produced when DSBs are prevented. However, in contrast to the outcome observed for *cra-1; spo-11* double mutants, γ-irradiating *cra-1; mre-11*, *cra-1; rad-51* and *cra-1; msh-5* double mutants did not rescue the extensive polymerization of SYP-1 along chromosome axes (Figure 8B, D, F). This indicates that the polymerization of SYP-1 along chromosome axes is coordinated with progression of recombination in the absence of CRA-1.

## Discussion

The novelty of our studies lies, first, in the identification and analysis of a conserved, yet previously uncharacterized protein required for SC formation in *C. elegans* and, second, in discovering that DSB formation and repair promote the polymerization of SC components along chromosome axes in the absence of CRA-1 function in *C. elegans*. Therefore, these studies provide new biological insights into the mechanism driving the polymerization of SC components and establish the foundation for the investigation of the role of CRA-1 in those organisms where SC formation is DSB-dependent.

### Regulation of Proper SC Assembly Between Homologs is CRA-1-Mediated

In *cra-1* mutants, homologous chromosomes can identify each other, as is made evident by the presence of PC-mediated, synapsis-independent pairing [4],[12] and the low incidence of nonhomologous pairing. However, homologous pairing interactions are not stabilized, resulting in a lack of chiasmata. These defects stem from defects in SC assembly. Specifically, while axis morphogenesis is normal in *cra-1* mutants compared to wild type, loading of central region components is aberrant, occurring in variable levels and along mostly unsynapsed chromosomes as opposed to only between paired homologs. Western blot analysis indicates that this is not due to a reduction in SYP-2 protein levels in *cra-1* mutants (Figure S6). Although the antibodies currently available preclude a similar assessment for either SYP-1 or SYP-3, their reduction is unlikely given their interdependency with SYP-2 ([5],[38], K. Schild-Prüfert and M. Colaiacovo, unpublished results). Therefore, our data suggest that in wild type, CRA-1 plays a critical role in preventing central region components from associating with isolated axes and in promoting their association between paired homologous axes.

**Figure 9:**
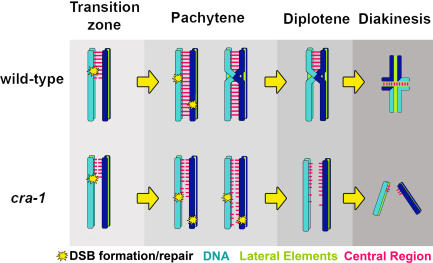
A Model for the Role of CRA-1 in Chromosome Synapsis. CRA-1 is not required for the establishment of homologous pairing or the nucleation of the central region components of the SC in the transition zone. However, it is essential for regulating the subsequent proper polymerization of the SC such that it bridges paired homologous axes in wild type. This is required for proper progression of meiotic recombination, resulting in the maturation of crossing over events during pachytene and chiasma formation. In *cra-1* mutants, the nucleation of central region components occurs, but their subsequent polymerization is deregulated resulting in their uneven assembly along chromosome axes. This polymerization depends on DSB formation and repair, but does not frequently result in a structure that bridges homologous axes. Therefore, interhomolog recombination resulting in crossing over events fails and chiasmata are not observed in diakinesis oocytes. For simplicity, the nucleation events are presented starting at one end of the chromosome and proceeding unidirectionally.

Several observations suggest that while CRA-1 is dispensable for the nucleation of central region components onto chromosomes, it is essential for their subsequent spreading. First, both genetic and cytological analysis place CRA-1 downstream of the lateral element protein HIM-3 and the central region proteins SYP-2 and SYP-3. Therefore, supporting a role for CRA-1 downstream from lateral element formation and the nucleation of central region components. Second, whereas extensive and continuous localization of SYP-1 is observed between nonhomologous chromosomes in *htp-1* mutants, spreading of SYP-1 is no longer observed in *cra-1; htp-1* double mutants and instead is replaced by foci and short stretches of chromatin-associated SYP-1. Furthermore, the mostly additive phenotypes of the *htp-1* and *cra-1* mutations fit into a model in which both proteins contribute to the proper assembly of the SC, but regulate two distinct aspects of this process and are not interdependent for these functions. HTP-1 was shown to be essential for the establishment of homologous pairing and coordinating pairing and synapsis [7],[8], whereas CRA-1 is essential for proper spreading of the SC once a homolog is identified and nucleation of the central region has started. Therefore our combined genetic and cytological evidence support a model where CRA-1 plays a role in SC assembly, downstream of homolog recognition (Figure 9).

How does CRA-1 promote SC assembly following nucleation? One possibility is that CRA-1 drives spreading of central region components by acting as a structural component of the SC. Alternatively, it may only perform a regulatory function or have both a structural and regulatory role during SC formation. The lack of antibodies that detect a discrete localization for CRA-1 on whole mounted gonads precludes us from determining whether CRA-1 plays a structural role as an additional SC central region component. However, based on multiple lines of evidence, we favor the idea that it primarily plays a regulatory role in coordinating SC assembly to occur between homologous chromosomes, thereby stabilizing their interactions. First, CRA-1 lacks the secondary structure that is a hallmark of central region components of the SC in many species: an extended coiled-coil domain flanked by globular domains [1],[2]. Instead, it contains a TPR motif, which is frequently observed in proteins that regulate biological processes such as transcription or cell cycle progression [27],[28],[29]. Second, immunofluorescence studies indicated that all null mutants for genes encoding structural components of the central region of the SC in *C. elegans* lack any chromosomal association of SYP-1, SYP-2 or SYP-3 [4],[5],[6]. Moreover, they lack any observable SC by TEM analysis [4],[5],[6]. In contrast, in *cra-1* null mutants the SYP proteins are observed localizing throughout chromosome axes during pachytene and SC stretches are occasionally observed. Furthermore, the combined set of changes observed in chromosome morphogenesis in *cra-1* mutants, such as the partially extended transition zone accompanied by unsynapsed chromosomes, have not been previously observed in any mutants of lateral/central structural components of the SC.

### Lack of CRA-1 Reveals a DSB-Dependent Pathway Promoting the Assembly of SC Components

In fungi (budding yeast, *Sordaria* and *Coprinus*), plants such as *Arabidopsis*, and mice, DSBs are needed to promote SC assembly. Mutants lacking Spo11 protein cannot properly assemble the central region of the SC, and therefore chromosome synapsis is perturbed [19],[20],[21],[41],[42],[43]. In *S. cerevisiae*, the formation of the synapsis initiation complex (SIC), comprised of proteins such as Zip1, Zip2, and Zip3, is dependent on recombination, as in *spo11* mutants, Zip2 and Zip3, which are required to recruit Zip1, fail to localize to chromosomes [44],[45]. In addition, the SIC proteins colocalize and/or physically interact with a number of recombination proteins [44],[45],[46], suggesting that SC assembly is physically intertwined with recombination. Indeed, analysis of a series of *spo11* missense mutants generating varying degrees of DSBs points to a correlation between the number of crossing over events and the levels of Zip3 foci, suggesting that DSB repair via homologous recombination facilitates synapsis by stabilizing pairing interactions through crossover intermediates [47]. The tight correlation between chromosome synapsis and sites of crossover formation is further supported by the observation that SICs are essential for maintaining normal levels of recombination [44],[45]. However, recombination may not be the only mechanism involved in promoting SC assembly, as some level of SC formation is still observed in *spo11* mutants in these organisms: in *S. cerevisiae*, 11% of cells arrested in pachytene carry normal SC structures [48], 48% of mice oocytes show extensive, although mainly nonhomologous, synapsis [20] and synapsis occurs in up to 10% of the total axial element length in *Arabidopsis* [49].

In contrast to yeast, plants and mammals, SC assembly is considered to be DSB-independent in both *C. elegans* and *Drosophila* females [22],[23]. Lack of DSB formation as a result of abrogating the function of the Spo11 protein in both worms and flies results in lack of chiasma formation; however, chromosome synapsis is indistinguishable from wild type [22],[23]. This may be due in part to how pairing interactions are established between homologous chromosomes in both worms and flies. In *C. elegans*, homolog recognition is pairing center end-mediated and mutants that fail to form these pairing interactions lack SC formation [4],[12],[50],[51]. In *Drosophila*, pre-meiotic pairing interactions persist into meiosis [52],[53], therefore bypassing the requirement for DSB formation and a homologous recombination-mediated search for homology.

Through the analysis of CRA-1 function, we discovered that DSB formation and repair can promote the assembly of central region components of the SC along chromosome axes, but not the efficient formation of a tripartite SC structure, in *C. elegans*. Our analysis revealed that aggregates of SYP-1 are formed in *cra-1; spo-11* double mutants. However, the polymerization of SYP-1 along chromosome axes is rescued following the induction of exogenous DSBs in this mutant background. Moreover, lack of MRE-11, RAD-51 or MSH-5 function in a *cra-1* background also results in the formation of SYP-1 aggregates. However, these aggregates still persist after induction of exogenous DSBs. Given that chromosomes are perfectly synapsed in *spo-11, mre-11, rad-51* and *msh-5* mutants, altogether our studies implicate CRA-1 in promoting the DSB-independent formation of the tripartite SC in *C. elegans*. Previous studies, which examined double mutant combinations of *spo-11* and mutants that lack structural components of the central region of the SC, such as *syp-1*, *syp-2* and *syp-3* null [4],[5],[6], or mutants with severely reduced synapsis-independent pairing, such as *syp-3(me42)* ([38]; this study), would not have detected this process.

How does CRA-1 promote SC assembly in a DSB-independent manner? Our studies suggest that CRA-1 acts after the establishment of pairing, which is PC-mediated and synapsis-independent, by playing a direct role in regulating the efficiency and specificity of SC assembly. Therefore, CRA-1 promotes a polymerization of the SC that succeeds in connecting the axes of paired homologous chromosomes, thus bypassing a requirement for recombination intermediates to stabilize pairing interactions. Nevertheless, CRA-1 homologs are present in other multicellular organisms, yet these exhibit DSB-dependent SC assembly [20],[21],[49]. It is possible that these proteins have diverged in different manners that reflect their efficiency in promoting SC assembly, or that other proteins which act in the same pathway are present in one group of organisms and absent in the other. However, our studies suggest that it is likely that most organisms are capable of using both DSB-dependent and -independent mechanisms for SC assembly, but use these pathways to varying degrees. The degree to which a particular pathway is employed may correlate with one or more of the following: the strategy utilized in the sorting of homology as discussed earlier; the timing with which DSBs are generated with regard to SC formation; and the overall levels of DSBs. In organisms such as *Drosophila* and *C. elegans*, immunofluorescence studies revealed that early steps of recombination, such as DSB formation in the former [54] and strand invasion/exchange in the latter [5], occur later in meiotic prophase and at lower levels compared to organisms such as yeast and mice [21],[55],[56],[57],[58]. Therefore, these organisms may not rely on DSB-dependent mechanisms to guarantee synapsis and thus utilize mainly a DSB-independent pathway. Nevertheless, our data suggest that DSB-dependent mechanisms may be either activated or uncovered in certain *C. elegans* mutant backgrounds. These mechanisms may be potentially used in wild type cells when SC assembly is lagging, but are obscured in *spo-11, mre-11, rad-51 or msh-5* backgrounds, which still retain the either more efficient or earlier acting DSB-independent machinery. DSB-dependent assembly of central region components of the SC therefore becomes the prime mode of assembly once pairing interactions are initiated, but cannot be stabilized, as seen in *cra-1* mutants.

To conclude, our studies demonstrate that polymerization of SC subunits in *C. elegans* can be driven by DSB formation and require progression of DSB repair. However, CRA-1 plays a key role in promoting chromosome synapsis hence possibly previously obscuring the identification of a DSB-dependence in *C. elegans*. The analysis of CRA-1 related proteins in other organisms thus raises the possibility of further unanticipated insights into the relationship between synapsis and DSB formation and repair.

## Materials and Methods

### Genetics

All *C. elegans* strains were cultured at 20°C under standard conditions [59]. Bristol N2 worms were utilized as the wild type background. Hawaiian CB4856 wild type worms were utilized for assessing recombination frequencies. The following mutations and chromosome rearrangements were used ([3],[4],[5],[6],[7],[8],[60],[61],[62]; this work):

LGI: *syp-3(ok758, me42), hT2[bli-4(e937) qIs48] (I;III)*
LGIII: *cra-1(tm2144), nDf16, nDf17*
LGIV: *htp-1(me84), unc-44(e362), him-3(gk149), rad-51(lg8701), spo-11(ok79), msh-5(me23)*
LGV: *syp-2(ok307), unc-70(e493), mre-11(ok179), syp-1(me17).*

*cra-1* (open reading frame R13F6.10) was identified in a targeted functional genomics screen utilizing RNAi to identify meiotic candidates as described in [63], from among genes with germline-enriched expression presented in [64] (M. Colaiacovo unpublished results). The *tm2144* allele was generated by the *C. elegans* National Bioresource Project in Japan. It contains a 753 base pair out-of-frame deletion encompassing exons 3 to 5 and the majority of exon 6 of R13F6.10.

*tm2144* is a recessive *cra-1* allele. DAPI-stained germlines of *cra-1(tm2144)*/+ hermaphrodites, as well as the levels of both embryonic lethality and males observed among their progeny were indistinguishable from wild type (0% embryonic lethality and 0.03% male progeny; n = 2795).

Transheterozygotes for *tm2144* and either *nDf16* or *nDf17* were indistinguishable from *tm2144* homozygotes, as determined by examining their DAPI-stained germlines. Moreover, RNAi-mediated depletion of *cra-1* by either feeding or injection resulted in identical defects to those observed in *cra-1(tm2144)* homozygotes. Finally, whereas by Western blot analysis the 109 kDa predicted CRA-1 band was present in worm lysates (50 worms/20ul of lysis buffer prepared as in [32]) from either *cra-1(tm2144)*/*hT2[bli-4(e937) qIs48]* or N2 worms, it was absent in worm lysates prepared from *cra-1(tm2144)* homozygotes (Figure 1B). Taken together these results indicate that *cra-1(tm2144)* is a null.

### Determining Crossover Frequencies

Meiotic crossover frequencies were assayed utilizing single-nucleotide polymorphism (SNP) markers as in [65]. However, given the elevated embryonic lethality observed for *cra-1(tm2144)* mutants, recombination frequencies were assayed on single-egg instead of single-worm lysates prepared from eggs laid by *cra-1(tm2144)/+* and *cra-1(tm2144)/cra-1(tm2144)* hermaphrodites heterozygous for SNP markers on chromosomes V and X. The *cra-1(tm2144)/cra-1(tm2144)* hermaphrodites were generated by mating *cra-1(tm2144)*/*hT2[bli-4(e937) qIs48]* hermaphrodites from the Hawaiian strain CB4856 background to *cra-1(tm2144)*/*hT2[bli-4(e937) qIs48]* males from the Bristol (N2) background. Since *qIs48* is a chromosomally integrated transgene insertion of *ccEx9747* expressing GFP under control of the *myo-2*, *pes-10* and *ges-1* promoters (which drive GFP expression in the pharynx, embryos and intestine respectively; [66]), lack of GFP expression was used to identify cross-progeny animals. Homozygous *cra-1(tm2144)* cross-progeny was then mated to N2 males generating *cra-1(tm2144)/+* cross-progeny which was identified by PCR for presence of the wild type *cra-1* gene. Single-egg lysates from eggs laid by either *cra-1(tm2144)/+* or *cra-1(tm2144)/cra-1(tm2144)* hermaphrodites were utilized for PCR and *Dra*I restriction digests. The following *Dra*I SNP primers were utilized: pkP5076 and snp_Y17D7B for chromosome V and pkP6143 and uCE6-1554 for the X chromosome as in [67].

### FISH and Time Course Analysis of Chromosome Pairing

FISH probes were generated as in [23] from pooled cosmids kindly provided by the Sanger Center, and labeled with either fluorescein-12-dCTP (PerkinElmer) or Digoxigenin-11-dUTP (Roche). The following cosmids were used: D1037, ZC535, F21A9 (I, left); F14B11, F32A7 (I, right); F28C10, F57C12, F13C5, M6, M02A10, C02H7, T04G9, F25E2, C03F1, F56F10, ZC13 (X, left); T23E7, F20B4, F15G10, K09G11 (X, right).

Homologous pairing was monitored quantitatively as in [33], with FISH signals considered paired when separated by ≤0.75 µm. The average numbers of nuclei scored per zone (n) for wild type and *cra-1 (tm2144)* are as follows: zone 1 (n = 57), zone 2 (n = 66), zone 3 (n = 76), zone 4 (n = 76), zone 5 (n = 69), zone 6 (n = 66), and zone 7 (n = 60).

### Antibody Preparation, DAPI Analysis, and Immunostaining

Rabbit α-CRA-1 antibody was generated using a HIS-tagged fusion protein expressed from plasmid pDEST 17 (Invitrogen) containing coding sequence corresponding to the first 400 amino acids of the CRA-1 protein. Animals were immunized and bled by Sigma-Genosys, The Woodlands, TX. Affinity purification of this antibody was performed as described in [68]. Specificity was demonstrated on Western blots where the CRA-1 antibody (1∶200 dilution) failed to detect CRA-1 protein on *cra-1* homozygous mutant lysates. Similar results were obtained using an antibody raised against a HIS tag fusion containing the last 400 amino acids of CRA-1 (data not shown).

DAPI staining, immunostaining and analysis of stained meiotic nuclei were performed as in [5]. Antibodies were used at the following dilutions: α-SYP-1 (1∶100), α-RAD-51 (1∶100), α-HTP-3 (1∶500), α-REC-8 (1∶100) and α-HIM-3 (1∶100). The secondary antibodies used were: Cy3 anti-rabbit, FITC anti-guinea-pig and FITC anti-mouse (Jackson Immunochemicals), each at 1∶100. Simultaneous antibody staining and FISH was performed on squashes as in [8]. Images presented are either high magnification (100X) or low magnification (60X) projections approximately halfway through 3D data stacks of whole nuclei, except for diakinesis images which encompass entire nuclei. Stacks of optical sections consisted of 15–30 0.2 µm slices acquired using the DeltaVision wide-field fluorescence microscope system (Applied Precision).

### Time Course Analysis for RAD-51 Foci

Quantitation of RAD-51 foci was performed for all seven zones composing the germline as in [5]. The average number of nuclei scored per zone (n) for wild type and *cra-1(tm2144)* were: zone 1 (n = 80), zone 2 (n = 91), zone 3 (n = 92), zone 4 (n = 69), zone 5 (n = 67), zone 6 (n = 65) and zone 7 (n = 61).

### Germ Cell Apoptosis

Germ cell corpses were scored in adult hermaphrodites 20 hours post-L4 as in [40]. Between 36 and 75 gonad arms were scored for each genotype. Statistical comparisons between genotypes were performed using the two-tailed Mann-Whitney test. *cra-1(tm2144)* differed significantly from wild type (p = <0.0001) and from *spo-11* (p = <0.0001), but did not differ significantly from *syp-1(me17)* (p = 0.7798).

### Electron Microscopy

Wild type and *cra-1(tm2144)* adult hermaphrodites (20–24 hr post-L4) were prepared for high pressure freezing as described in [4]. 100 nm-thick longitudinal sections of three wild type worms and two *cra-1* mutant worms were examined for the presence of SC in nuclei at the late pachytene region. Distances between electron-dense patches arranged in parallel were also measured (Figure 6). The range observed in wild type was 90 nm–125 nm (average distance = 118 nm). As a control, the distances between parallel electron-dense patches observed occasionally in *syp-3(me42)* pachytene nuclei were measured (n = 31; 2 worms). The range observed in *syp-3(me42)* was 46 nm–86 nm (average distance = 66 nm), consistent with our previous observation that SC stretches are not present in this mutant [6]. An SC stretch was scored as such in *cra-1* nuclei only when the distances between electron-dense patches were within the range observed in wild type.

### γ-Irradiation

Adult hermaphrodites (24 hours post-L4) were treated with a dose of 5000 rads of γ-rays from a ^137^Cs source. Their gonads were dissected, fixed and immunostained with α−SYP-1 antibody, as described above, 1, 4, 8 and16 hours following irradiation.

## Supporting Information

Figure S1 Localization of Central Region Components in Nuclei in the Transition Zone Region in *cra-1* Mutant Gonads. (A–D) High magnification images of nuclei from the transition zone immunostained with anti-HTP-3 (red) to visualize the lateral element and anti-SYP-1 (green) to visualize the central region, presented with (A–B) and without the DAPI signal (C–D). While in wild type, SYP-1 colocalizes to chromosomal regions containing HTP-3, in *cra-1* mutants, SYP-1 mostly encapsulates the chromosomes to which HTP-3 is extensively localized. Bars, 2 µm.
(6.33 MB DOC)

Figure S2 DSB Formation Rescues SYP-1 Polymerization Along Chromosome Axes in *cra-1* Mutants (A–F) High magnification images of DAPI-stained diakinesis nuclei immunostained with anti-SYP-1. (A) In wild type, SYP-1 localizes to the mid-section of all six bivalents. (B) In *spo-11* mutants, SYP-1 is observed localizing discontinuously on univalents presumably between sister-chromatids. (C) In *cra-1* mutants, SYP-1 is localized between sister-chromatids mostly as a single dot at the terminal end of the univalents. Arrow indicates a bivalent in which SYP-1 assembles at the mid-section, as observed in wild type. (D) In *cra-1; spo-11* mutants, a SYP1 aggregate is observed (indicated by arrow). This aggregate is mostly associated with chromosomes and, occasionally, is connected to a short patch of SYP-1 observed between sister-chromatids (inset depicts the SYP-1 signal at a higher magnification). (E) 8 hours post-γirradiation, the aggregates start to become less apparent in *cra-1; spo-11* mutants (a residual aggregate is apparent on one univalent indicated by the arrow). (F) By 16 hours post-γirradiation, *cra-1; spo-11* irradiated mutants completely revert to the SYP-1 localization observed in *cra-1* mutants. (G) Partial depletion of *cra-1* by RNAi in *spo-11* mutants results in formation of SYP-1 aggregates in pachytene nuclei. Inset depicts the SYP-1 signal alone for the nucleus indicated by the arrow. Bars, 2 µm.
(3.31 MB TIF)

Figure S3 CRA-1 Acts Downstream of Central Region Components of the SC (A–F) High magnification images of late pachytene nuclei co-stained with DAPI (blue) and anti-SYP-1 antibody (red). (A–B) In a *cra-1; him-3* double mutant, SYP-1 dots are observed instead of the SYP-1 patches observed in *him-3* mutants. (C–D) In *cra-1; htp-1* double mutants, SYP-1 nucleation is not impaired, however, an additive effect is observed as SYP-1 staining along chromosomes is far less extensive then observed in either single mutant. (E–F) *syp-3; cra-1* double mutants are indistinguishable from *syp-3* mutants with respect to chromosome morphogenesis and impaired SYP-1 localization, indicating that the *cra-1* phenotypes are dependent on the presence of the SYP complex. Insets correspond to nuclei indicated by arrows where detection thresholds for anti-SYP-1 signal were significantly lowered emphasizing lack of SYP-1 staining. Bars, 2 µm.
(8.35 MB TIF)

Figure S4 Aggregates of Central Region Components Observed in *cra-1; spo-11* Are Not a Result of Impaired Axis Morphogenesis (A–D) High magnification images of *cra-1; spo-11* mid-pachytene nuclei immunostained with anti-HTP-3 (green) to visualize the lateral element and anti-SYP-1 (red) to visualize the central region. While HTP-3 localizes continuously along chromosome axes, indicating that axis morphogenesis is normal in this background, the SYP-1 signal is mostly concentrated in a single aggregate per nucleus. Bars, 2 µm.
(1.23 MB TIF)

Figure S5 Polymerization of Central Region Components Along Chromosome Axes in *syp-3(me42); spo-11* Mutants High magnification images of *syp-3(me42)* (A) and *syp-3(me42); spo-11* (B) mid-pachytene nuclei co-immunostained with DAPI (blue) and anti-SYP-1 (red). SYP-1 localization along chromosome axes in *syp-3(me42)* mutants is not affected by the *spo-11* mutation. Bars, 2 µm.
(2.98 MB TIF)

Figure S6 CRA-1 does not Control SC Assembly by Regulating SYP-2 Expression Levels Western blot analysis comparing wild type, *cra-1(tm2144)/hT2*, *syp-2* null and *cra-1(tm2144)* mutant lysates probed with anti-SYP-2 and anti-HDA-1 (loading control) antibodies. A wild type specific band corresponding to the expected 25 kDa SYP-2 protein is observed in *cra-1* mutants and is absent in *syp-2* null mutants. No alterations in SYP-2 levels are observed in *cra-1* mutants as compared to wild type and *cra-1(tm2144)/hT2* controls, indicating that misregulation of SC assembly is not the result of changes in SYP-2 expression levels.
(0.70 MB TIF)

Table S1 P-values from the Fisher's Exact Test performed for FISH data in Figure 3B comparing pairing levels between wild type and *cra-1* mutants.
(0.03 MB DOC)
