# Supplementary figures and images for: CRA-1 Uncovers a Double-Strand Break-Dependent Pathway Promoting the Assembly of Central Region Proteins on Chromosome Axes During C. elegans Meiosis
Source: PLoS Genet. 2008 Jun 6;4(6):e1000088. doi: 10.1371/journal.pgen.1000088 (PMC2408554; doi:10.1371/journal.pgen.1000088)

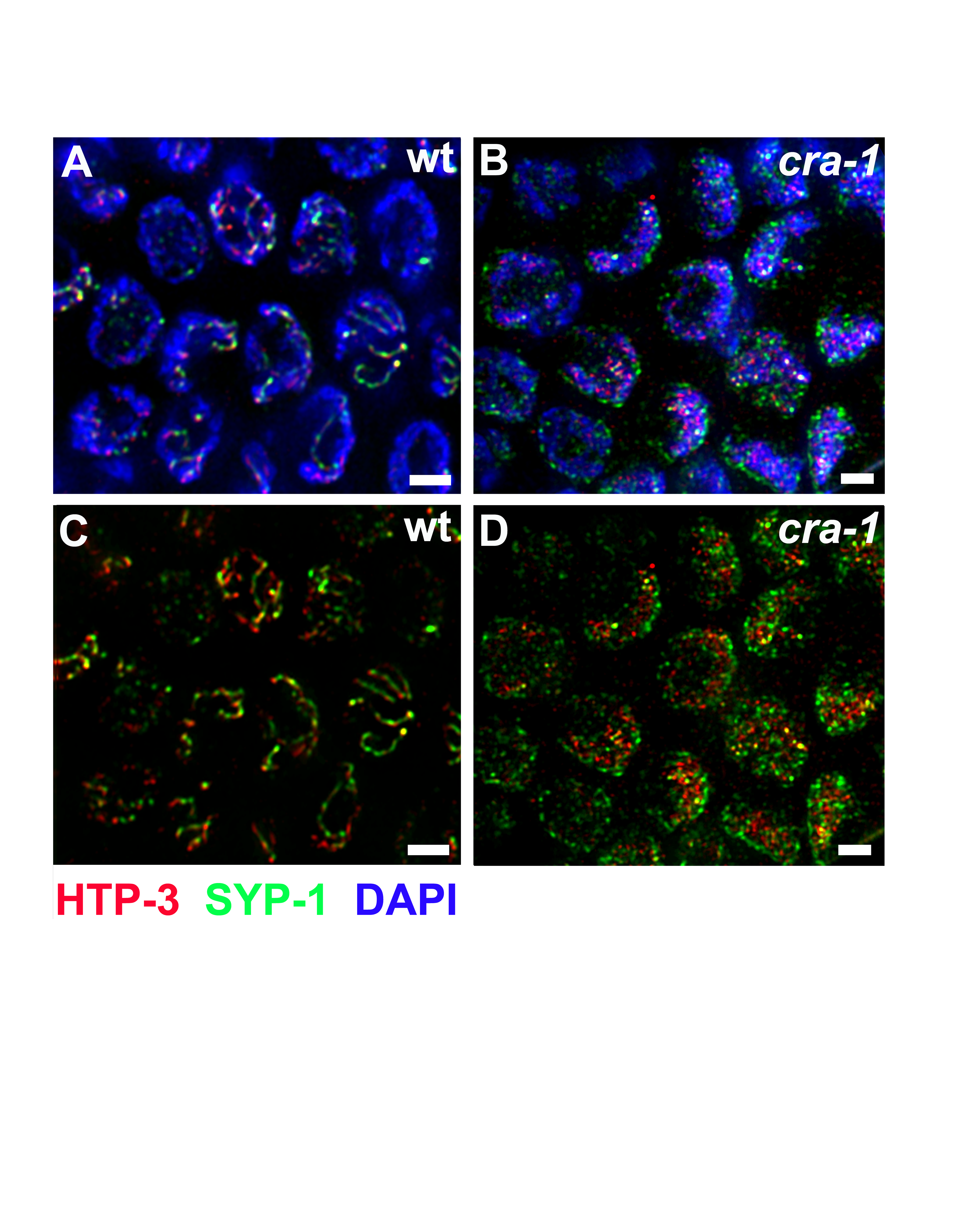

Supplement: Figure S1 — Localization of Central Region Components in Nuclei in the Transition Zone Region in cra-1 Mutant Gonads. (A–D) High magnification images of nuclei from the transition zone immunostained with anti-HTP-3 (red) to visualize the lateral element and anti-SYP-1 (green) to visualize the central region, presented with (A–B) and without the DAPI signal (C–D). While in wild type, SYP-1 colocalizes to chromosomal regions containing HTP-3, in cra-1 mutants, SYP-1 mostly encapsulates the chromosomes to which HTP-3 is extensively localized. Bars, 2 µm. (6.33 MB DOC) [file pgen.1000088.s001.tif]

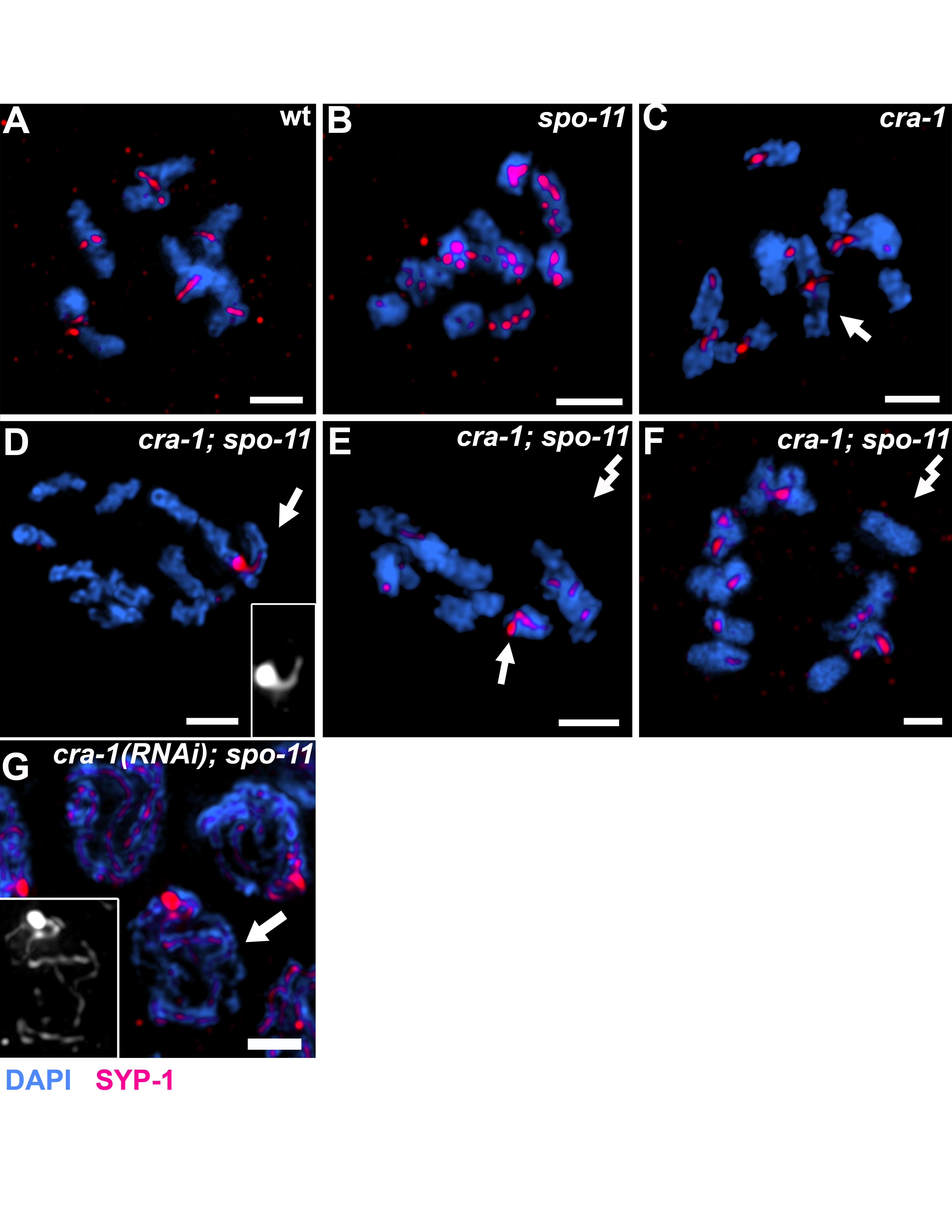

Supplement: Figure S2 — DSB Formation Rescues SYP-1 Polymerization Along Chromosome Axes in cra-1 Mutants (A–F) High magnification images of DAPI-stained diakinesis nuclei immunostained with anti-SYP-1. (A) In wild type, SYP-1 localizes to the mid-section of all six bivalents. (B) In spo-11 mutants, SYP-1 is observed localizing discontinuously on univalents presumably between sister-chromatids. (C) In cra-1 mutants, SYP-1 is localized between sister-chromatids mostly as a single dot at the terminal end of the univalents. Arrow indicates a bivalent in which SYP-1 assembles at the mid-section, as observed in wild type. (D) In cra-1; spo-11 mutants, a SYP1 aggregate is observed (indicated by arrow). This aggregate is mostly associated with chromosomes and, occasionally, is connected to a short patch of SYP-1 observed between sister-chromatids (inset depicts the SYP-1 signal at a higher magnification). (E) 8 hours post-γirradiation, the aggregates start to become less apparent in cra-1; spo-11 mutants (a residual aggregate is apparent on one univalent indicated by the arrow). (F) By 16 hours post-γirradiation, cra-1; spo-11 irradiated mutants completely revert to the SYP-1 localization observed in cra-1 mutants. (G) Partial depletion of cra-1 by RNAi in spo-11 mutants results in formation of SYP-1 aggregates in pachytene nuclei. Inset depicts the SYP-1 signal alone for the nucleus indicated by the arrow. Bars, 2 µm. (3.31 MB TIF) [file pgen.1000088.s002.tif]

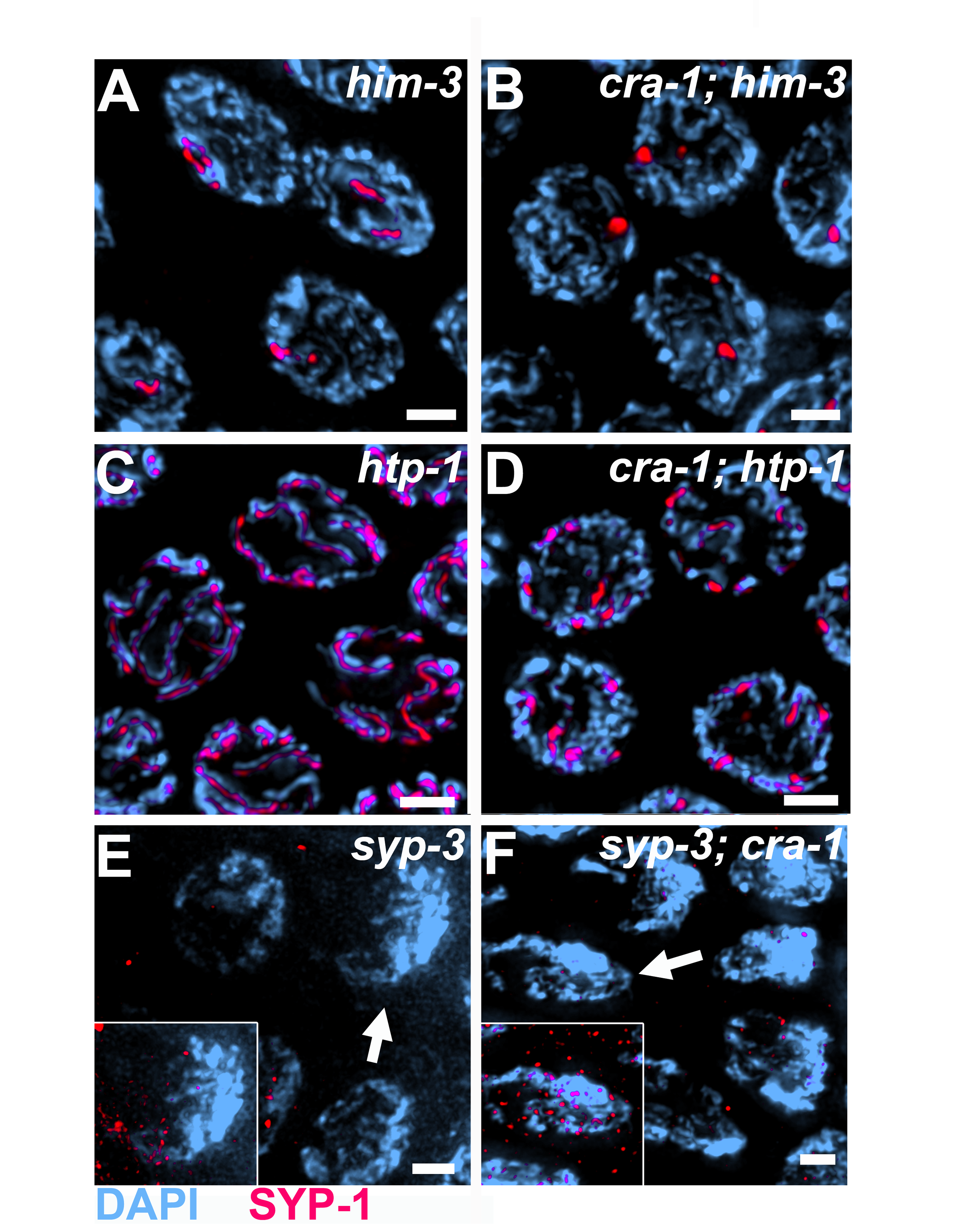

Supplement: Figure S3 — CRA-1 Acts Downstream of Central Region Components of the SC (A–F) High magnification images of late pachytene nuclei co-stained with DAPI (blue) and anti-SYP-1 antibody (red). (A–B) In a cra-1; him-3 double mutant, SYP-1 dots are observed instead of the SYP-1 patches observed in him-3 mutants. (C–D) In cra-1; htp-1 double mutants, SYP-1 nucleation is not impaired, however, an additive effect is observed as SYP-1 staining along chromosomes is far less extensive then observed in either single mutant. (E–F) syp-3; cra-1 double mutants are indistinguishable from syp-3 mutants with respect to chromosome morphogenesis and impaired SYP-1 localization, indicating that the cra-1 phenotypes are dependent on the presence of the SYP complex. Insets correspond to nuclei indicated by arrows where detection thresholds for anti-SYP-1 signal were significantly lowered emphasizing lack of SYP-1 staining. Bars, 2 µm. (8.35 MB TIF) [file pgen.1000088.s003.tif]

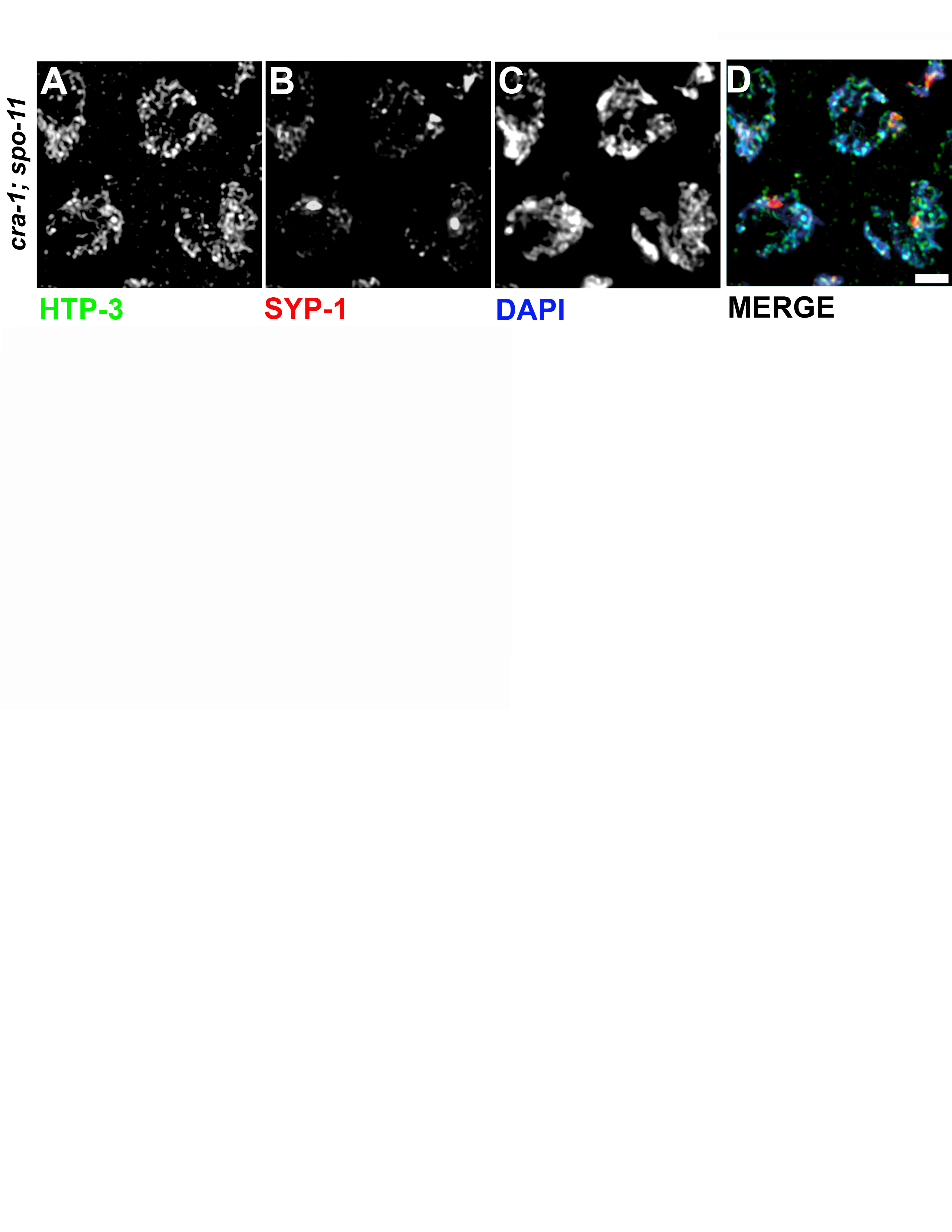

Supplement: Figure S4 — Aggregates of Central Region Components Observed in cra-1; spo-11 Are Not a Result of Impaired Axis Morphogenesis (A–D) High magnification images of cra-1; spo-11 mid-pachytene nuclei immunostained with anti-HTP-3 (green) to visualize the lateral element and anti-SYP-1 (red) to visualize the central region. While HTP-3 localizes continuously along chromosome axes, indicating that axis morphogenesis is normal in this background, the SYP-1 signal is mostly concentrated in a single aggregate per nucleus. Bars, 2 µm. (1.23 MB TIF) [file pgen.1000088.s004.tif]

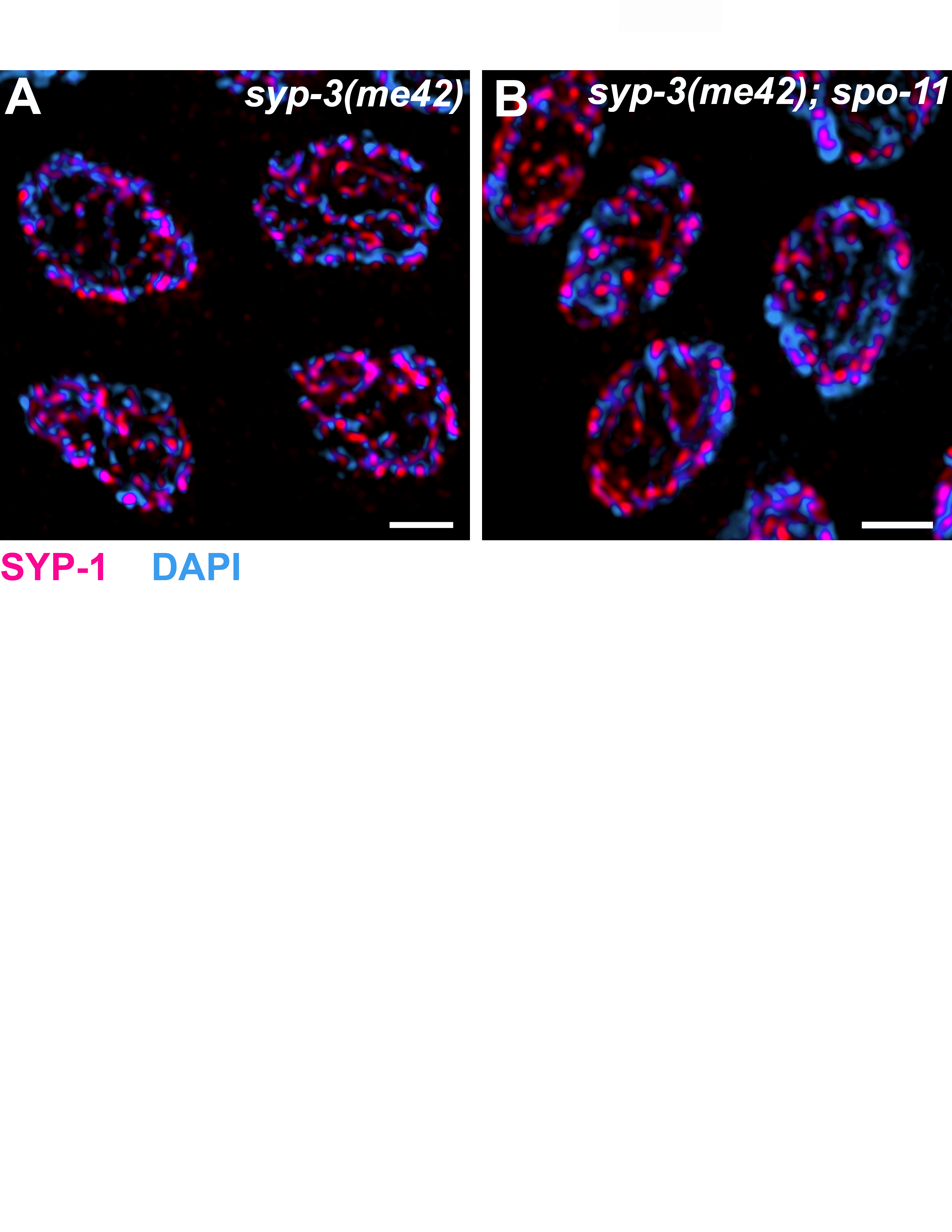

Supplement: Figure S5 — Polymerization of Central Region Components Along Chromosome Axes in syp-3(me42); spo-11 Mutants High magnification images of syp-3(me42) (A) and syp-3(me42); spo-11 (B) mid-pachytene nuclei co-immunostained with DAPI (blue) and anti-SYP-1 (red). SYP-1 localization along chromosome axes in syp-3(me42) mutants is not affected by the spo-11 mutation. Bars, 2 µm. (2.98 MB TIF) [file pgen.1000088.s005.tif]

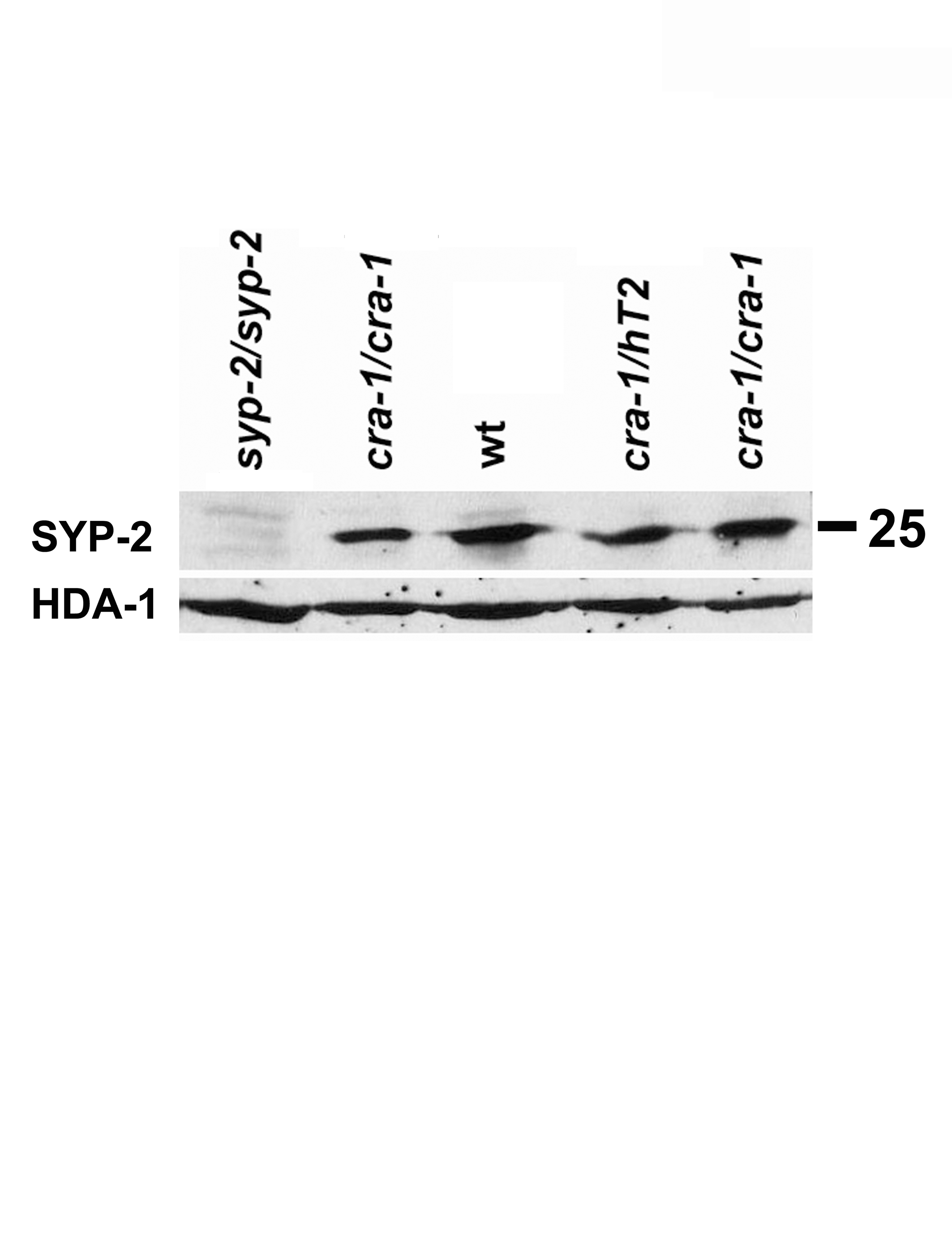

Supplement: Figure S6 — CRA-1 does not Control SC Assembly by Regulating SYP-2 Expression Levels Western blot analysis comparing wild type, cra-1(tm2144)/hT2, syp-2 null and cra-1(tm2144) mutant lysates probed with anti-SYP-2 and anti-HDA-1 (loading control) antibodies. A wild type specific band corresponding to the expected 25 kDa SYP-2 protein is observed in cra-1 mutants and is absent in syp-2 null mutants. No alterations in SYP-2 levels are observed in cra-1 mutants as compared to wild type and cra-1(tm2144)/hT2 controls, indicating that misregulation of SC assembly is not the result of changes in SYP-2 expression levels. (0.70 MB TIF) [file pgen.1000088.s006.tif]
