# Supplementary material for: CRA-1 Uncovers a Double-Strand Break-Dependent Pathway Promoting the Assembly of Central Region Proteins on Chromosome Axes During C. elegans Meiosis
Source: PLoS Genet. 2008 Jun 6;4(6):e1000088. doi: 10.1371/journal.pgen.1000088 (PMC2408554; doi:10.1371/journal.pgen.1000088)
Supplement: Table S1 — P-values from the Fisher's Exact Test performed for FISH data in Figure 3B comparing pairing levels between wild type and cra-1 mutants. (0.03 MB DOC) [file pgen.1000088.s007.doc]

**________________________________________________________________________**

**Table S1.** P-values from the Fisher's Exact Test performed for FISH data in Figure 3B comparing pairing levels between wild type and *cra-1* mutants

|  | **Zone 1** | **Zone 2** | **Zone 3** | **Zone 4** | **Zone 5** | **Zone 6** | **Zone 7** |
| --- | --- | --- | --- | --- | --- | --- | --- |
| I PC | 0.4110 | 0.1363 | <0.0001 | <0.0001 | <0.0001 | <0.0001 | <0.0001 |
| X PC | 0.5714 | 0.3738 | 0.1080 | 0.0425 | 0.0801 | 0.0001 | <0.0001 |
| I NPC | NA | 0.2997 | <0.0001 | <0.0001 | <0.0001 | <0.0001 | <0.0001 |
| X NPC | 1 | 0.2997 | <0.0001 | <0.0001 | <0.0001 | <0.0001 | <0.0001 |

Zones are as indicated in Figure 3A.

The significance of the pairing levels was tested by the Fisher's Exact Test (two-tailed p value and 95% confidence intervals) using the InStat software (Graphpad).

NA = Not Applicable (0% pairing levels were observed for both wt and *cra-1*)
